# Gender Incongruence in Danish Youth (GenDa): A Protocol for a Retrospective Cohort Study of Danish Children and Adolescents Referred to a National Gender Identity Service

**DOI:** 10.3390/jcm13226658

**Published:** 2024-11-06

**Authors:** Nanna Ravnborg, Mohsin Aslam, Pernille Badsberg Norup, Jonas Vrublovsky Tingsgård, Anne Katrine Pagsberg, Mette Ewers Haahr, Katharina M. Main, Annamaria Giraldi

**Affiliations:** 1 Department of Growth and Reproduction, Copenhagen University Hospital—Rigshospitalet, 2100 Copenhagen, Denmark; 2 International Centre for Research and Research Training in Endocrine Disruption of Male Reproduction and Child Health (EDMaRC), Rigshospitalet and University of Copenhagen, 2100 Copenhagen, Denmark; 3 Sexological Clinic, Mental Health Centre Copenhagen, Mental Health Services, 2200 Capital Region of Denmark, Denmark; 4 Child and Adolescent Mental Health Center, Mental Health Services, 2100 Capital Region of Denmark, Denmark; 5 Department of Clinical Medicine, University of Copenhagen, 2200 Copenhagen, Denmark

**Keywords:** gender identity, gender incongruence, transgender, health, mental health, hormone therapy

## Abstract

**Background/Objectives** In recent years, the national Gender Identity Service for individuals under 18 years of age in Denmark has seen a considerable increase in referrals of youngsters during puberty. Given this development, it is important to deepen our understanding of the characteristics of contemporary youngsters seeking help for gender incongruence. This understanding can serve as the foundation for improving current treatment regimens by ensuring optimal individual assessment and care. In this study, we aim to describe the sociodemographic characteristics, health profiles, and treatment trajectories in detail, as well as any changes in these characteristics, of all transgender and gender-diverse youngsters referred to the Gender Identity Service in Denmark from 2016 through 2022. **Methods**: This is a retrospective observational study of a national cohort comprising all individuals under 18 years of age referred to the Danish Gender Identity Service from 1 January 2016 to 1 January 2023. We will use data from medical records obtained at routine visits from the first assessment through repeated visits. Data on demographics, physical and mental health profiles, and information regarding gender identity will be collected and analyzed. The characteristics of those individuals who progressed to hormone therapy will be compared to those who did not. **Results:** This study aims to enhance our understanding of the characteristics and needs of contemporary youngsters with gender identity issues. **Conclusions**: The scientific evidence for the assessment and treatment of gender incongruence in youngsters is limited. The characteristics of youngsters seeking healthcare for gender incongruence today may differ from earlier.

## 1. Introduction

In recent years, a substantial increase in referrals of youngsters with gender incongruence to gender identity services has been seen worldwide [1,2,3,4]. Gender incongruence is defined as a discrepancy between an individual’s gender identity and their sex assigned at birth [5]. This incongruence may stem from the traditional binary view of gender in many Western cultures [6]. At the gender identity services, youngsters and their families seek counseling and hormone therapy to alleviate any distress caused by gender incongruence. In this protocol, individuals who identify as male and were assigned female sex at birth are described as transmasculine youngsters, and individuals who identify as female and were assigned male sex at birth are described as transfeminine youngsters.

In Denmark, more than 1300 youngsters were referred to the national service between 2016 and 2022 with increasing referral numbers over time [7]. The increase in referrals is particularly notable among individuals older than 11 years. Since 2016, there has been a predominance of transmasculine youngsters (approximately 70%) among the referred individuals. This pattern is observed in youngsters aged 11–17 years, while it is not present in younger children. A similar pattern has been described in several other countries, including the Netherlands, the United Kingdom, Sweden, Norway, Italy, Canada, and the United States of America [4,8]. In the adult population, an increase in referrals is also reported from many countries but without such a skewed sex ratio [9,10]. Studies have suggested a higher level of psychiatric morbidity along with higher suicide rates among transgender and gender-diverse (TGD) individuals [11,12]. The most frequently reported psychiatric diagnoses among TGD individuals include neurodiversity and mental health issues such as autism spectrum disorder, attention-deficit/hyperactivity disorder (ADHD), depression, and anxiety [4,13,14]. These patterns differ from descriptions in early studies [15].

In Denmark, endocrine treatment follows the current clinical practice guidelines from the Endocrine Society, from 2017. It encompasses two main treatment principles: puberty suppression using gonadotropin-releasing hormone analogs (GnRHa) and gender-affirming hormone therapy (GAHT) with sex steroids, i.e., testosterone or estradiol [16].

Puberty suppression in transmasculine individuals leads to some regression of breast tissue growth in the early pubertal stages and cessation of menses. In transfeminine individuals, further virilization is prevented, and testicular volume may decrease. Consequently, spermatogenesis and follicle maturation are arrested [16,17,18]. Puberty suppression with GnRHa is considered reversible, allowing the resumption of pubertal development, and is well tolerated in children with precocious puberty [16,18,19,20]. This pause in physical changes prevents the progression of incongruence between body appearance and gender identity, providing a period for further clarification before starting testosterone or estradiol treatment. However, potential adverse effects include headaches, mood changes, sterile abscesses from injections, hot flushes, and fatigue [16,18,20,21,22].

Masculinizing testosterone therapy leads to cessation of menses, increase in muscle mass and strength, fat redistribution, voice deepening, growth of facial and body hair, oily skin or acne, clitoral enlargement, and increased libido. It can lead to male pattern baldness and may affect fertility [16,22,23,24,25,26,27]. Additionally, breakthrough bleeding [28] and pelvic pain [29] have been reported during testosterone therapy. Feminizing estrogen and antiandrogen treatment leads to the development of breast tissue, fat redistribution, some decrease in facial and body hair, clearer and softer skin, reduction of libido, decrease in muscle mass and strength, reduction of testicular volume and sperm quality [16,22,23,26,27,30]. While puberty suppression is reversible when stopping treatment, most effects of GAHT are irreversible, such as breast development and body hair growth. 

Research related to gender incongruence in youngsters is still sparse, and current evidence is predominantly based on studies that are more than five years old. Emerging evidence, however, suggests potential health risks associated with hormone therapy. This includes the effects of GnRHa on bone health and brain development [31], the cardiovascular risks associated with GAHT, such as dyslipidemia and venous thromboembolism, and long-term risks, such as cancer and osteopenia [16,22,27]. Furthermore, there is a knowledge gap regarding the effects of hormone therapy on youngsters, including its impact on height, the development of secondary sex characteristics, and changes in body composition [32].

TGD individuals have a higher risk of being subject to stigmatization and harassment compared to their cisgender peers [33,34,35]. Hormone therapy is reported to improve psychosocial functioning among help-seeking TGD youngsters [36,37], and although the impact on quality of life varies, it generally trends towards improvement [38,39]. However, there is a lack of knowledge about the long-term effects, and small sample sizes in reported studies limit firm conclusions. Investigating gender incongruence and the psychosocial challenges, including minority stress, faced by TGD individuals can help improve the identification of the support and treatment they need. Additionally, evaluating the impact of counseling and hormone therapy can enhance best-practice care. Thus, it becomes increasingly imperative to explore the characteristics and needs of the young TGD group [32].

In recent years, individuals who revise their decision to undergo GAHT over time, especially those who regret the irreversible physical changes associated with GAHT [40,41], have received more attention. Current knowledge of reasons to stop hormone therapy or to detransition to the birth-assigned gender is very limited, and the number of individuals changing their treatment trajectory over time is likely underreported [32,42,43].

In many countries, including Denmark, detransitions and debates regarding treatment have led to the precaution of postponing decisions on hormone therapy when gender incongruence has been brief. This is especially true when there are concerns about the stability of the experienced gender identity [44,45,46], as fluctuations and experimentation with gender expression are common among youngsters [47,48]. However, this precaution may have consequences as it could diminish the degree to which the individual can socially pass as the experienced gender, especially for transfeminine individuals. This may exacerbate minority stress and worsen mental health problems [49,50].

The Danish health care program for individuals under 18 years of age experiencing gender incongruence was established in 2016 as a multidisciplinary team (MDT) involving Sexological Clinic, Mental Health Services Centre Copenhagen, Rigshospitalet; Department of Growth and Reproduction, Rigshospitalet; and Child and Adolescent Mental Health Centre, Mental Health Services Capital Region, Bispebjerg, all affiliated with the University of Copenhagen. It was based on international experiences and the so-called Dutch protocol [51,52]. However, it has been proposed that the Dutch protocol may need refinement for the contemporary population seeking medical care [32,53] as the composition of the group of help-seeking youngsters has changed in the last 10–15 years. This has raised concerns due to the lack of evidence-based guidelines for assessing and treating this contemporary group. The Danish cohort of TGD youngsters has not yet been described, and systematic long-term outcome studies on health, safety, and well-being are limited internationally [32,44]. Given this lack of data, along with the controversies surrounding hormone therapy [32,54], the debate over detransitions, and the changed composition of the referred TGD group, there is a pressing need for structured, large-scale data collection and research focused on treatment outcomes, detransitions, and profiles of youngsters who do not receive GAHT. There is also a growing interest in reporting experiences from healthcare centers worldwide [55,56]. This study aims to address this knowledge gap, provide evidence to inform best practices for medical and psychological treatment, and update treatment guidelines by characterizing all referred Danish TGD youngsters from 2016 to 2022. Additionally, we are planning a protocol for follow-up studies of this national cohort.

### 1.1. Assessment

In Denmark, the assessment and treatment program at the National Gender Identity Service, including semen cryopreservation, is part of the National Health Service [7]. Individuals under the age of 18 years with gender identity concerns can be directly referred by general practitioners or other health care professionals without prior evaluation.

Before the age of 10 years, parents or guardians are offered reflective consultations to help them support their child. After the age of 10 years, the youngster is offered either a full assessment program or psychosocial support following an initial screening visit, during which the gender incongruence and its duration are assessed. The full assessment program is then initiated if indicated.

The psychosocial support program consists of two or three in-person sessions with the youngster that include reflections on gender, body image, and sexuality. After these sessions, the youngster is concluded from the service.

The full assessment program comprises a minimum of five in-person consultations at the Sexological Clinic, aimed at exploring the youngster’s history of gender identity concerns, body image, wishes for treatment, development of sexuality, and overall well-being. The youngster’s parental support and peer network are also assessed. At the Child and Adolescent Mental Health Centre, a psychiatric screening for any psychopathology, an evaluation of cognitive function, and an assessment of the global level of functioning are conducted simultaneously. Based on the results of the psychiatric screening, additional psychological testing is then performed using well-validated tests, with supporting psychometric studies published between 1938 and 2023. The family is also seen at the pediatric endocrinologic department for information on hormone therapy possibilities, their effects, and potential risks. When the full assessment is completed, an MDT conference is held to discuss a treatment plan, including a decision on whether puberty blockers or GAHT can be offered. If no consensus is achieved, further counseling sessions may be necessary. Additional MDT conferences are held each time the treatment plan needs to be revised, such as for the initiation of GAHT after puberty suppression with GnRHa monotherapy or deciding on referral to mastectomy at the age of 18 years. Consultations addressing gender identity, well-being, and psychosocial effects of treatment continue after the initiation of hormone therapy [57].

**Figure A1:**
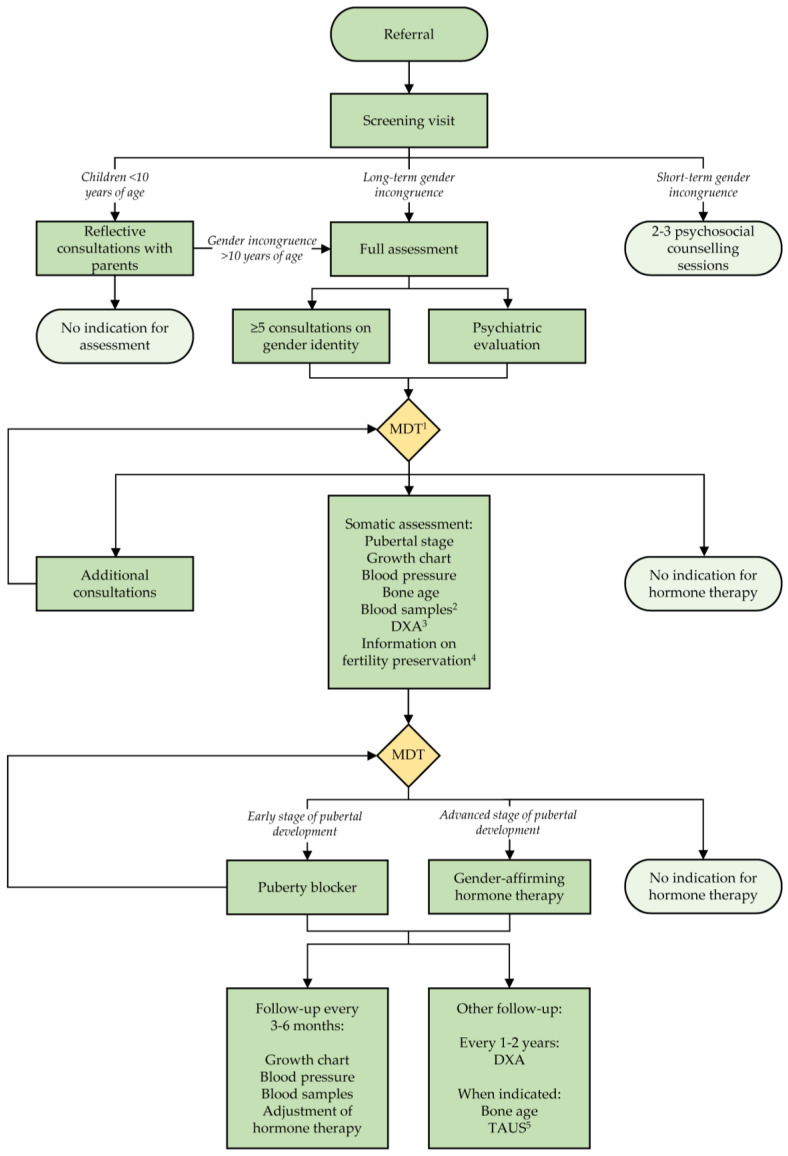
Assessment and treatment pathways for transgender and gender-diverse youngsters referred to the Gender Identity Service in Denmark. 1. *Multidisciplinary team conference. 2. Reproductive and adrenal hormones, sex steroids, growth factors, thyroid hormones, lipids, liver and kidney parameters, vitamin D, hematocrit, and hemoglobin. 3. Dual-energy X-ray absorption. 4. Semen cryopreservation is offered to transfeminine individuals before hormone therapy after individual assessment. 5. Transabdominal ultrasound.*

See Figure A1 for assessment and treatment pathways.

### 1.2. Endocrine Treatment

Puberty suppression with GnRHa can be initiated from Tanner stage 2 [58,59,60]. GnRHa is given as depot injections. GAHT encompasses estradiol in transfeminine youngsters and testosterone in transmasculine youngsters. Sex steroid treatment doses are titrated over 6 to 12 months to reach adult serum levels of estradiol or testosterone. Testosterone therapy is initiated with daily transdermal gel applications. When adult doses are reached, intramuscular depot testosterone injections are offered as an alternative. Estradiol is administered with transdermal patches, gel, or tablets, depending on personal preferences. When testosterone concentrations in transmasculine youngsters reach adult levels, GnRHa is discontinued. GnRHa is continued parallel to estradiol therapy in transfeminine youngsters until referral to adult services.

Before hormone therapy, a somatic assessment is performed, i.e., a growth chart, including bone age and growth velocity; pubertal staging, if relevant; blood pressure; routine blood samples; and whole-body Dual-energy X-ray Absorptiometry (DXA) to evaluate body composition and bone mineral density. Routine blood samples consist of reproductive and adrenal hormones, sex steroids, growth factors, vitamin D, lipids, liver and kidney parameters, hematocrit, hemoglobin, and thyroid hormones, measured by liquid chromatography-tandem mass spectrometry (LC-MS/MS) or immunoassays. Semen cryopreservation is offered to transfeminine youngsters if biologically possible. Transabdominal ultrasound (TAUS) of the internal genitalia for transmasculine youngsters is performed on indications such as breakthrough bleedings but was performed routinely after one year of testosterone therapy until 2021. Routine visits every three to six months consist of blood samples (hormones and safety parameters), growth assessment if relevant, weight, blood pressure, and exploration of treatment results, effects, and side effects. DXA scans are performed every two years; bone age is repeated if indicated. Serum, plasma, and DNA are collected for a biobank at each clinical visit.

At 18 years of age, the youngsters are referred to continued care in adult gender services at one of three centers in Denmark [57].

### 1.3. Aims

This study aims to:

1. describe the sociodemographic background of TGD individuals under 18 years of age referred for evaluation of gender incongruence
2. describe the development of gender identity and incongruence
3. investigate the impact of social transitioning on gender identity (social transition is defined in this protocol as living in accordance with one’s experienced gender identity)
4. describe the development of sexuality
5. describe the physical health characteristics, including short-term and long-term clinical effects, safety, and efficacy of hormone therapy
6. describe the mental health characteristics, such as the prevalence of psychiatric disorders and the global level of functioning
7. describe the prevalence of substance abuse, self-harm, and suicidal ideation
8. explore the well-being of youngsters offered hormone therapy until their discharge from the team at the age of 18 years
9. describe the number, characteristics, and reasons for youngsters who detransitioned, discontinued and/or regretted hormone therapy before being discharged from the team at the age of 18 years
10. describe trends and changes in the diagnostic process and treatment trajectories in the study period.

## 2. Materials and Methods

### 2.1. Experimental Design

This protocol describes a retrospective observational study conducted in a national cohort from a single tertiary medical center. The study population includes all individuals under 18 years of age who were referred to and attended at least one appointment at the Gender Identity Service in Copenhagen from 1 January 2016 to 1 January 2023. There are no exclusion criteria.

The study collects longitudinal data from medical records on the experiences with and development of gender identity, gender incongruence, and sexuality for all individuals in the study population. Furthermore, the study gathers information on the social context of the youngsters, such as demographics, socioeconomic status, family circumstances, school attendance, and social networks. Mental health data collection encompasses psychiatric symptoms and diagnoses, results of psychological tests, suicidal thoughts, substance abuse, and global level of functioning. Physical health data collection includes hormone therapy type, its effects and side effects, anthropometry, DXA scans, blood samples, and semen cryopreservation. Data on discontinuation of hormone therapy and treatment trajectories are also collected. See Appendix A Table A1 and Table A2 for details on data collection.

### 2.2. Ethics and Legal Permissions

The study has obtained approval from the Danish Patient Safety Authority and the Health Capital Region Team for Medical Records Research, respectively, to access the medical records of all eligible individuals with a waiver of individual consent (3-3013-3117/1, R-21045474, and R-23014626).

Data are securely stored within a web-based database using the Research Electronic Data Capture (REDCap) tools [61,62] (LTS version 13.7.14) hosted at the Capital Region of Denmark. All data in this project are handled in accordance with the European and National Acts on Processing of Personal Data. The project is registered in the Capital Region of Denmark according to the European Union General Data Protection Regulation (GDPR) art. 30 (p-2019-230).

The project on the individuals receiving hormone therapy, including biobank samples, is approved by the local ethics committee (H-18050607) and requires individual consent. Additional scientific studies outside this scope will need new approval by the Ethical Board and Data Protection Authority who then decide whether a new individual consent is required depending on the nature of the new project. Inclusion in this part of the study is entirely voluntary. Consent can be withdrawn at any time by the parent or guardian or by the youngsters themselves from age 15 years onwards without providing any explanation. This includes the option to request the destruction of personal data and biological samples.

Participation or non-participation in any part of the project must not (by law) affect the medical healthcare related to gender incongruence.

### 2.3. Data Source and Procedure

Every visit to one of the three departments of the Gender Identity Service is routinely registered in the electronic medical records. Data collection and data entry to the REDCap database is undertaken by a designated team of researchers who are trained systematically and undergo repetitive interobserver validation assessments.

### 2.4. Analysis Plan

Data will be extracted from the REDCap database and analyzed in pseudo-anonymized datasets. Descriptive statistics will be used for sociodemographic characteristics, physical and mental health profiles, and treatment trajectories, i.e., mean (SD), median (IQ range), and frequencies (proportion). The data will be divided into subgroups according to whether hormone therapy is offered before the age of 18 years and whether they are referred to the Gender Identity Service before or after the onset of puberty. The subgroups will be compared according to sex assigned at birth, age of onset of gender incongruence, social transition, as well as sociodemographic background, mental health profiles, and global level of functioning. Physical health profiles of the youngsters in the hormone therapy subgroup will be compared to normative age- and relevant gender-matched reference populations. Group comparisons will be made using the Student’s *t*-test and Mann–Whitney/Wilcoxon tests as appropriate. Changes over time will be analyzed using time trend analysis for related data and paired *t*-tests or mixed model analysis. Regression models will investigate confounding factors related to treatment/no treatment outcomes and basic characteristics. If appropriate, analyses will be reported by year, i.e., trends in referral patterns and decision algorithms.

## 3. Expected Results

By conducting a retrospective observational study, we can characterize the Danish TGD youngsters over seven years. Due to our unbiased national cohort, we can provide unique and important insights into this group. Additionally, studies on gender incongruence deepen our understanding of the complexities of gender identity and expression, thereby increasing awareness and understanding of gender diversity within society and among healthcare professionals [6]. Based on our findings, we aim to optimize and tailor assessment and treatment protocols by increasing our knowledge, thus preventing harm and improving the well-being of TGD youngsters seeking healthcare.

## 4. Strengths and Limitations

This retrospective study has the considerable strength of being based on a nationwide single-center cohort, which limits selection bias from participation agreement, drop-out, regional referral, recruitment, and assessment practices. 

The data are collected systematically over a seven-year study period, allowing us to compare contemporary Danish youngsters with populations from early Dutch studies. Additionally, we can examine short-term treatment outcomes and how assessment and treatment practices have evolved during this time in Denmark.

There are no exclusion criteria for this study, as we aim to describe the entire national population of all youngsters referred to the Gender Identity Service. This approach allows us to gather a wide range of data and identify trends that might have been missed in a more restricted sample. By including all individuals in our cohort, we also reduce the risk of bias.

By describing an unselected group of youngsters with gender incongruence, we can characterize a spectrum of gender identities, including those who are not binary transgender. Additionally, we can include youngsters with gender incongruence who do not receive GAHT, which is valuable to the scientific and societal debate.

This study will provide unique initial data on our cohort, generating hypotheses and laying a foundation for future research, including potential longitudinal follow-up into adulthood.

Assessment and treatment at the Gender Identity Service were conducted as part of routine National Health Care. There will be some missing data since the data are collected retrospectively from medical records. Additionally, the list of variables and measures is limited to what can be extracted from the medical records.

The study population is limited to individuals who have attended at least one appointment at the Gender Identity Service, as there is no available data from the medical records on individuals who were referred to the Gender Identity Service but were not accepted.

## 5. Patients’ Involvement and Dissemination Strategy

The study findings will be published in peer-reviewed journals and presented at international conferences and scientific meetings. Furthermore, study findings will be presented to relevant healthcare providers, at the National Board of Health, NGOs, and patient and parent groups.

## 6. Study Registration

Clinical Trials.gov NCT06573177. Registered on 27 August 2024.

## Appendix A

**Table A1:** List of assessment variables to be extracted for analysis for youngsters who attended at least one appointment at the Danish Gender Identity Service from 2016 through 2022.

Assessment Variables	Value
Demographics	
Date/age at referral	Date (DD-MM-YYYY)/age in years (number)
Date/age at first appointment	Date (DD-MM-YYYY)/age in years (number)
Birth country	Denmark/other
Preterm birth	Yes/no
Adoption	Yes/no
Sex assigned at birth	Male/female
Family	
Family composition	Parents together/parents not together
Living situation	Parents/step-parents/foster parents/institution/other
Custody	Shared/mother/father/other
Siblings	Number
Twin	Yes/no
Conflicts at home	Many/few
Close relationship with parent/primary caregiver	Yes/no
Parents	
Socioeconomic status	High/mid/low
Occupation/support	Employed/student/unemployed/maternity leave/sick leave/on public welfare/on pension
Type of occupation	High-level professionals and CEOs/mid-level professionals and managers/blue-collar workers and service industry employees/unskilled laborers
Diseases	Somatic/psychiatric
School	
Type of school	Public school/special abilities school/treatment school/online school/not in school
School attendance	High/mid/low frequency
Change in school	Yes/no Number
Academic level	High/mid/low
Social skills (with classmates)	High/mid/low
Friends	
Type of friendship	Online/in real life/both
Rate of contacts	Daily/weekly/monthly/yearly
Closeness	Yes/no
Spare time	Alone/with friends/with family
Loneliness	Yes/no
Gender identity	
Gender identity	Masculine/feminine/non-binary/other
Age at onset of gender incongruence	Age in years (number) Before/during/after the onset of puberty
Social transition	Yes/partial/no
Age at social transitioning	Before/during/after the onset of puberty
Gender expression	Masculine/feminine/other
Body dysphoria	Yes/no
Family support	Yes/partial/no
Treatment wishes and expectations	Hormone therapy/operation/counseling/legal sex change/other
Fertility wishes	Biological children/adoption/no children/in doubt
Sexuality	
Sexual orientation	Male/female/non-binary/all genders/other
Romantic relationships	Yes, current/yes, prior/no
Sexual experiences	Yes/no
Number of sexual partners (lifetime)	Number
Threats and bullying	
Threats	Yes/no
Bullying	Yes/no
Violence	Yes/no
Childhood abuse	Yes/no
Sexual assault	Yes/no
Self-harm and suicidal ideation	
Self-harm	Yes, current/yes, prior/no
Suicidal thoughts	Yes, current/yes, prior/no
Suicide attempts	Yes, current/yes, prior/no
Self-reported psychiatric symptoms	
Traits of psychiatric disease	Yes, current/yes, prior/no
Psychiatric morbidity	
Prior registered diagnoses	Yes/no
Consultation at a child and adolescent psychiatric emergency room	Yes/no
Admitted to a child and adolescent psychiatric hospital	Yes/no
Pharmacological psychiatric treatment	Yes/no
Substance abuse	
Alcohol abuse	Yes, current/yes, prior/no
Smoking	Yes, current/yes, prior/no
Drug abuse	Yes, current/yes, prior/no
Self-medication	
Testosterone/estrogen/other	Yes/no
**Psychiatric Evaluation Variables**	**Value**
Psychiatric morbidity	
Registered diagnoses	Yes/no Diagnosis codes
Pregnancy and birth	Normal/abnormal
Early psychomotor development	Normal/abnormal
Psychological tests and evaluations (on indication)	K-SADS (Kiddie Schedule for Affective Disorders and Schizophrenia) [63] PSE (Present State Examination) [64] SRS-2 (Social Responsiveness Scale) [65] BRIEF (Behavior Rating Inventory of Executive Function) [66] ADHD-RS (ADHD-Rating Scale) [67] WISC 4/5 (Wechsler Intelligence Scale for Children) [68] WAIS 4 (Wechsler Adult Intelligence Scale) [69] ADOS (Autism Diagnostic Observation Schedule) [70] ADI-R (Autism Diagnostic Interview-Revised) [71] ABAS (Adaptive Behavior Assessment System) [72] SCID (Structured Clinical Interview for DSM-5 Personality Disorder) [73] Roberts [74] Rorschach [75] C-GAS (Children’s Global Assessment Scale) [76] GAPD (Global Assessment of Psychosocial Disability) [77]
**Treatment Trajectory Variables**	**Value**
Healthcare use	
Total number of visits	Number
Date of first visit	Date (DD-MM-YYYY)
Date of last visit	Date (DD-MM-YYYY)
Outcome of assessment (multidisciplinary conference decision on GAHT)	Yes/no/other
Date of discharge from Gender Identity Service	Date (DD-MM-YYYY)
**Visits Variables**	**Value**
Repeated measures	
Changes in any listed variable	

**Table A2:** List of treatment variables to be extracted for analysis for youngsters who attended at least one appointment at the Danish Gender Identity Service from 2016 through 2022.

Treatment Variables	Value
Basis, Department of Growth and Reproduction	
Date/age at referral for information only	Date (DD-MM-YYYY)/age in years (number)
Date/age at visit for information only	Date (DD-MM-YYYY)/age in years (number)
Date/age at referral for hormone therapy	Date (DD-MM-YYYY)/age in years (number)
Date/age at first visit for hormone therapy	Date (DD-MM-YYYY)/age in years (number)
Somatic diagnoses	Yes/no Diagnosis code
D-vitamin supplement	Yes/no Dose Date of initiation (DD-MM-YYYY) Date of cessation (DD-MM-YYYY)
Medication	Asthma medicine/allergy medicine/sleeping medicine/dermatological medicine/biological medicine/other
**Gonadotropin-releasing hormone analogs (GnRHa) Variables**	**Value**
Date/age at initiation	Date (DD-MM-YYYY)/age in years (number)
Administration route and dose	11.25 mg/12 weeks subcutaneously (SC) 11.25 mg/12 weeks intramuscularly (IM) 22.5 mg/24 weeks IM
Date/age of GnRHa cessation	Date (DD-MM-YYYY)/age in years (number)
GnRHa—repeated measures	
Date of modification of GnRHa therapy	Date (DD-MM-YYYY)
Modified dose or administration route	Dose IM
Modified interval	Weeks (number)
Side effects of GnRHa	Yes/no Headache/nausea/hot flashes/mood swings/disturbed sleep
**Gender-affirming hormone therapy (GAHT) variables**	**Value**
Date/age of initiation	Date (DD-MM-YYYY)/age in years (number)
Start of administration route	Injections IM/gel transdermal (TD)/tablets orally (PO)/patch TD
Start dose	Registered respective to the choice of drug
Discontinuation of GAHT	Discontinued by TGD-individual/discontinued by the health care team
Reasons for discontinuation of GAHT	Regret/needs more time/side effects/other
Date/age of discontinuation of all GAHT	Date (DD-MM-YYYY)/age in years (number)
GAHT, transmasculine youngsters—repeated measures	
Date of modification	Date (DD-MM-YYYY)
Modified dose or administration route	Dose IM/TD
Side effects of GAHT	Yes/no Oily skin/acne/rash/itch/breast tenderness/tenderness after injections/lower abdominal pain/other
Voice deepening	Yes/no
GAHT, transfeminine youngsters—repeated measures	
Date of modification	Date (DD-MM-YYYY)
Modified dose or administration route	Dose PO/TD
Side effects of GAHT	Yes/no Oily skin/acne/rash/itch/breast tenderness/tenderness after injections/lower abdominal pain/other
Breast development	Tanner stage B1/B2/B3/B4/B5
**Transmasculine youngsters variables**	**Value**
Puberty development a.m. Tanner	Tanner stage B1/B2/B3/B4/B5 Tanner stage PH1/PH2/PH3/PH4/PH5/PH6 Full puberty
**Transabdominal ultrasound scans—repeated measures**	
Date of ultrasound	Date (DD-MM-YYYY)
Uterus size	Length × width × depth (mm)
Cervix length	Number (mm)
Endometrium thickness	Number (mm)
Endometrial layers	1/2/3
Right/left ovary—size	Length × width × depth (mm)
Right/left ovary—number of small follicles	Not visualized/none/few/moderate/many
Right/left ovary—biggest follicle	Number (mm)
**Transfeminine youngsters variables**	**Value**
Puberty development a.m. Tanner	Tanner stage G1/G2/G3/G4/G5 Tanner stage PH1/PH2/PH3/PH4/PH5/PH6 Full puberty
Testis volume right/left	Volume (mL)
Opting for semen cryopreservation	Yes/no
**Semen cryopreservation—repeated measures**	
Date/age of semen collection	Date (DD-MM-YYYY)/age in years (number)
Azoospermia	Yes/no
Volume	Number (mL)
Concentration	Number (mill/mL)
Progressive motility spermatozoa	Percentage (%)
Non-progressive motility spermatozoa	Percentage (%)
Immotile spermatozoa	Percentage (%)
Did not complete semen collection	True/false
**Visits—repeated measures variables**	**Value**
Date of visit	Date (DD-MM-YYYY)
Blood pressure	Systolic blood pressure (number) Diastolic blood pressure (number)
**Other clinical data variables**	**Value**
Dual-energy X-ray absorptiometry (DXA) scan	Bone mineral density (BMD) (g/cm^2^) + Z-scores Total body fat percentage (%) Regional body fat percentage (%) Lean body mass (LBM) (g)
Growth chart	Height (cm) + standard deviation score (SDS) Weight (kg) + SDS Growth velocity (cm/year) + SDS Predicted adult height (cm) + SDS Target height (cm) + SDS
Bone age (a.m. Greulich-Pyle, BoneXpert)	Age in years (number)
Routine blood samples	Reproductive hormones Growth factors Thyroid hormones Vitamin D Lipids Liver-kidney function tests Hematocrit Hemoglobin
Biobank	Serum, plasma, DNA
